# Development of Patient Education Materials for Total Joint Replacement During an International Surgical Brigade

**DOI:** 10.5435/JAAOSGlobal-D-20-00074

**Published:** 2020-10-14

**Authors:** Derek S. Stenquist, Lauren V. Ready, Roya Ghazinouri, Carolyn Beagan, Aliesha Wisdom, Jeffrey N. Katz

**Affiliations:** From the Harvard Medical School, Boston, MA (Dr. Stenquist, Dr. Katz); the Harvard Combined Orthopaedic Residency Program, Massachusetts General Hospital, Boston, MA (Dr. Stenquist); the Orthopedic and Arthritis Center for Outcomes Research, Brigham and Women's Hospital, Boston, MA (Dr. Stenquist, Ready, Dr. Ghazinouri, Dr. Katz); the Department of Medicine, Center for Healthcare Delivery Sciences, Brigham and Women's Hospital (Dr. Ghazinouri), Boston, MA; the Departments of Rehabilitation Services, Physical Therapy (Dr. Ghazinouri, Beagan), Brigham and Women's Hospital, Boston, MA; the Department of Orthopedic Surgery (Dr. Ghazinouri, Wisdom, Dr. Katz), Brigham and Women's Hospital, Boston, MA; the Department of Epidemiology, Harvard School of Public Health, Boston, MA (Dr. Katz); and the Division of Rheumatology, Immunology, and Allergy, Brigham and Women's Hospital, Boston, MA (Dr. Katz).

## Abstract

Temporary brigade trips to deliver international surgical care are increasingly common. For the purposes of this work, we use the term brigade to describe self-contained short-term medical or surgical mission trips where healthcare professionals are brought in from foreign regions to provide care to an underserved population. Many brigade programs have begun to collect and publish data on outcomes and complications, but few have examined their own patient education practices.

**Methods:**

We used evidence-based readability and suitability analyses along with patient interviews to develop improved patient education materials for a total joint replacement surgical brigade in the Dominican Republic.

**Results:**

Existing patient education materials required an eighth grade reading level and lacked suitability based on the principles of educational theory. The redesigned materials required fifth grade reading skills or less and had superior suitability. Pilot testing with patients from the target population suggested that the materials were appealing and appropriate.

**Conclusions:**

Patient education may play an important role in optimizing outcomes in the setting of medical or surgical brigades where resources and access to follow-up care are limited. More research is needed to bring attention to the importance of patient education during brigades, and programs should work with patients to develop educational materials that are suitable and effective.

Short-term trips by medical professionals from high-income countries (HICs) to low- and middle-income countries (LMICs) to provide nonemergent medical and surgical care are increasingly common.^1-3^ Tens of thousands of patients receive care through such international medical or surgical “brigades” each year,^3,4^ yet there are no universal minimum operating standards for these programs.^2,3,5,6^ Although many brigade programs now publish data on outcomes and complications,^2,4,7,8,9,10,11,12,13,14^ whether their patients have access to appropriate patient educational materials is not known. We examined the issue of patient education in the context of a total joint replacement (TJR) surgical brigade.

Musculoskeletal disorders are a leading cause of global years lived with disability, and developing countries have experienced a threefold greater increase in the burden of musculoskeletal disorders than developed countries in the past two decades.^15,16^ This trend is expected to continue with population growth and improved longevity in the developing world, resulting in negative socioeconomic consequences for national economies.^15,17^ Osteoarthritis (OA) of the knee and hip is particularly prevalent and causes notable functional impairment and morbidity.^18,19^ In response, surgeons from HICs have begun to offer total knee and hip replacement surgery to underserved patients in LMICs during annual surgical brigades to address the burden of surgical disease and to build local capacity for delivering musculoskeletal surgical care.^12^

Knee and hip replacement surgeries are cost-effective procedures^20-22^ that offer patients with advanced symptomatic arthritis relief from pain and improved functional status.^23-25^ However, TJR surgery is associated with considerable risk and requires rigorous follow-up and rehabilitation.^26,27,28,29,30^ Joint replacement programs in HICs often require patients to attend perioperative education programs in attempts to manage patient expectations, mitigate anxiety, and maximize outcomes.^31,32,33,34,35^ Patients in LMICs who receive TJR during international surgical brigades deserve the same access to health information as their counterparts in HICs. However, the transient nature of surgical brigades creates challenges for patient education and follow-up, which may leave patients at higher risk for suboptimal outcomes or complications.^2,36,37^

The Healthcare Consumer Bill of Rights and Responsibilities protects American patients' right to “accurate, easily understood information about their health, treatments, health plan, providers, and health care facilities,”^38^ and US healthcare facilities are required to provide access to appropriate patient education materials by the Joint Commission.^39,40^ There is no equivalent protection or accreditation process for temporary surgical brigades,^6^ and patient education materials for these settings have not been rigorously evaluated to our knowledge. As the number of global surgical brigades increases^3,41^ and surgical services are scaled up in LMICs,^42,43^ research is needed to explore challenges to and optimal modes of delivering perioperative information to patients with diverse backgrounds, education levels, and cultural contexts. We undertook a systematic evaluation of the patient education practices of a TJR surgical brigade that performs approximately 50 knee and hip replacements annually for underserved patients in the Dominican Republic with end-stage OA. We hypothesized that the existing patient education materials were not optimal for the target patient population and that improving the suitability of these materials would improve patient understanding of surgical risks and what to expect in the postoperative period. We used an iterative process and educational theory to develop improved patient education materials to bring attention to the importance of providing suitable patient education during international surgical brigades. We provide our methods and complete finalized materials (Appendix 1, http://links.lww.com/JG9/A85) for adaptation and use by other TJR brigades serving Spanish-speaking populations.

## Methods

### Readability and Suitability Estimates

We formally assessed the readability and suitability of patient education materials of a TJR brigade for this study. Readability is an objective measure of the reading skills necessary to understand a given document.^39^ Material written at a readability level above a reader's education level is unlikely to be comprehensible to that reader.^39^ There are many validated tools for estimating the readability of a document by the grade level.^39^ We chose the Fry readability graph because it is validated in English and Spanish, it can be administered manually, and it has been recommended by the Centers for Disease Control and Prevention (CDC).^44-46^ The use of the Fry readability graph to estimate readability has been described elsewhere.^44,47-49^

Suitability is an objective measure of the appropriateness of patient education materials for an adult audience based on educational theory.^49^ The Suitability Assessment of Materials (SAM) is a tool designed and validated for the systematic evaluation of print and illustrated materials.^49^ The SAM scale combines an assessment of many features of patient education materials such as content, print size and style, layout, concept density, readability (measured by the Fry readability graph), and images to determine a final suitability score.^49^ Proper use of the SAM to evaluate patient education materials has been described elsewhere.^49^

### Assessment of Existing Patient Education Materials

We undertook an interdisciplinary review of existing patient education materials designed by the US-based brigade team with a small group of providers including a physician, physical therapist, medical student, and research assistant. The group discussed goals and knowledge content for new patient education materials. We then used the Fry readability graph and SAM scale to estimate the readability and suitability of the existing Spanish-language patient education materials.

### Development of New Educational Materials

We adapted social marketing principles to provide structure for resource development. Social marketing in health promotion focuses on the needs of the target audience with the goal of influencing behavior change for the benefit of that target audience. The “4 P's” (product, price, place, promotion) approach was favored for its simplicity and its utility as a framework demonstrated in other peer-reviewed publications on patient education.^50^ Next, we developed a draft of the new patient education materials using an evidence-based resource from the CDC^44^ and published resources on designing health information materials for Latino/a audiences.^50,51,52,53,54^

Once the materials had been developed, we assessed them for suitability using the SAM in an iterative process before testing them with the target population. The initial developer of the new education materials rated them using the SAM and then made revisions. Next, a medical student with no affiliation with this study who is a native Spanish speaker undertook an independent assessment of the materials using the SAM. Additional revisions were made. Finally, a professor of medical Spanish who is a native Spanish speaker reviewed the materials for grammar and comprehension before testing with patients.

### Evaluation by Patients and Providers

**Figure 1:**
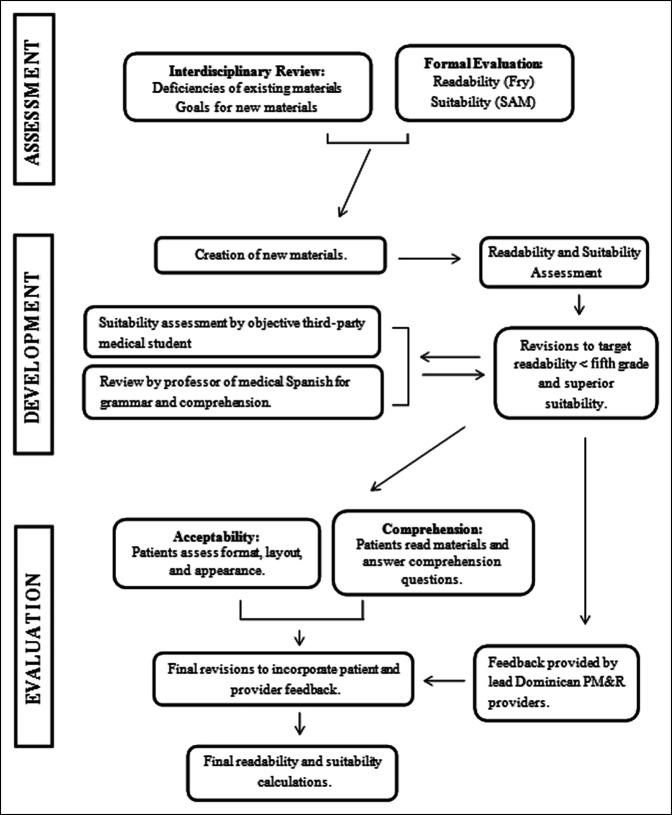
Flowchart summarizing the process of educational resource development and evaluation.

After initial development in the United States based on the existing materials, educational theory, and input from providers, we tested the materials with brigade patients. We provided the materials to all TJR surgery patients during the 2016 brigade and conducted individual interviews with a convenience sample of 20 patients to gather feedback. We administered a standardized questionnaire to these 20 patients to assess acceptability and comprehension and solicit suggestions for improvement. In addition, the materials were used by providers during the 2016 brigade to provide care for patients and feedback was solicited from the program's head Dominican Pain Medicine and Rehabilitation (PM&R) providers through e-mail. The process of assessment, development, and evaluation is summarized in Figure 1.

## Results

The interdisciplinary review identified three key areas of focus for the revised patient education materials: (1) must enhance patients' understanding of surgery, (2) must support patients in recovery, and (3) must improve the comprehension of discharge instructions. We revised the existing discharge instructions and physical therapy guidelines and developed a new frequently asked question (FAQ) sheet, thereby producing a document to address each area: (1) FAQ about the surgical procedure and postoperative milestones, (2) a comprehensive physical activity and physical therapy guide, and (3) revised discharge instructions.

### The Social Marketing Approach

The 4 P's provided a framework for development of the new educational materials within the unique context of a surgical brigade. Our “product” was the knowledge delivered to patients through the new education materials. We used an evidence-based approach to assessing readability and suitability of our product to minimize the “price” (the emotional and time costs required to acquire the knowledge) for our patients. Consideration of patients' emotional mindset and cultural context (“place”) was crucial to the development of appropriate materials.^52,55^ Finally, we chose written materials supplemented by the provider's instructions as the mode of “promotion” or knowledge delivery because of the busy schedule of a surgical brigade. This choice was supported by the studies of Hispanic patients' health education preferences.^51,53,54^

### Patient Interviews

Demographic characteristics for the patients included in the convenience sample are provided in Table 1. All patients were interviewed in their hospital rooms during the preoperative period or no more than 2 days postoperatively. Ten patients (50%) elected to have the materials read to them. Six of these patients (30%) stated that they were too tired from surgery or did not have glasses with them, whereas four patients (20%) admitted that they could not read.

**Table 1:** Demographics for the Group of Patients Who Participated in Interviews to Provide Feedback on Revised Materials

Demographics	
No. of participants	20
Age (mean [range])	59 (45-82)
Female	18 (90%)
Male	2 (10%)
Educational attainment	
Less than secondary school	9 (45%)
Secondary school but did not graduate	2 (10%)
Graduated from secondary school or beyond	9 (45%)
Illiterate (self-reported)	4 (20%)
Illiterate patients reporting literate family member at home	4 (100%)

### Acceptability

Overall, 16 of 20 patients interviewed (80%) felt that the materials were “fine” or “great” in their current form and did not suggest any changes. Four patients (20%) made specific suggestions regarding language, formatting, and content, which were incorporated into the final version of the materials. These included suggestions to use more colloquial language, clarify the purpose of a medication, include more images, and emphasize compliance with medications and physical therapy. In response to specific questions, 19 patients (95%) felt that the materials were good and not too “busy.” Fourteen patients (70%) stated that they or their family would use the discharge pamphlet to write down questions; the remaining patients suggested we provide more room for writing dates of future appointments or questions. Eighteen patients (90%) stated that they or their family would use the discharge pamphlet to remember their follow-up appointments. Eighteen patients (90%) stated that they would use the physical therapy guide and discharge pamphlet to remember their physical therapy exercises.

### Comprehension

Patients were asked to read or listen to the section on warning signs from the discharge pamphlet and the section on daily activity milestones from the FAQ sheet. Patients were then asked brief comprehension questions regarding the information provided. Overall, patients demonstrated better comprehension of the warning signs than activity milestones, with at least 85% of patients answering correctly for all questions regarding potentially life-threatening or limb-threatening situations. However, more than half of patients (55%) stated that they would call the doctor immediately if they experienced mild pain after performing physical therapy exercises (an incorrect response). For activity milestones, patients performed well on questions about when they would be able to climb a flight of stairs or return to work after surgery but struggled with questions about when they would be able to shower, return to sexual activity, and drive a car.

### Provider Feedback

Overall, Dominican PM&R providers felt that patients' awareness and understanding of their physical therapy had improved with the use of the revised patient education materials in comparison to previous years. Suggestions for improvement of the materials included providing specific recommendations for patients undergoing bilateral joint replacements and more orientation to patients about turning on their axis. They also noted that patient education procedures could be strengthened by including more detailed physical therapy education before surgery.

### Readability and Suitability of New Patient Education Materials

**Figure 2:**
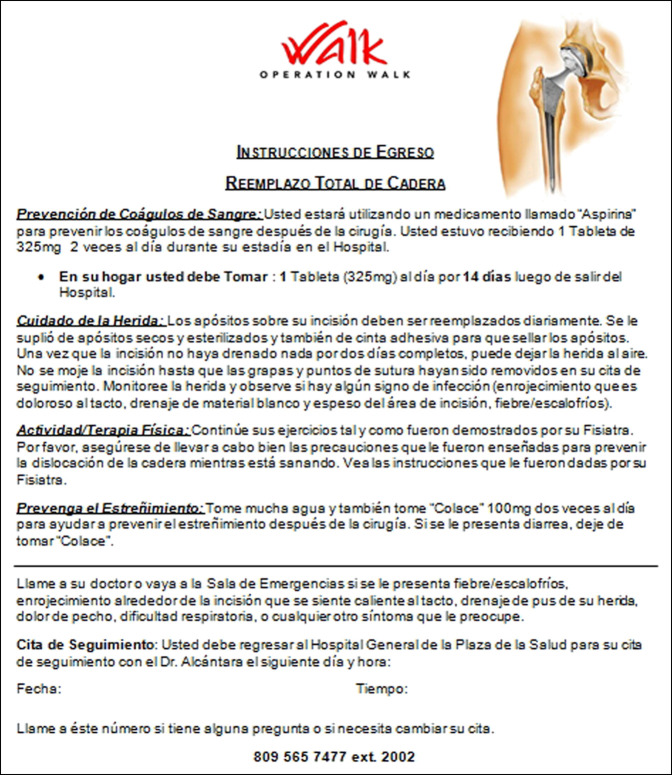
Photograph showing the original brigade discharge material for total hip replacement (THR).

**Figure 3:**
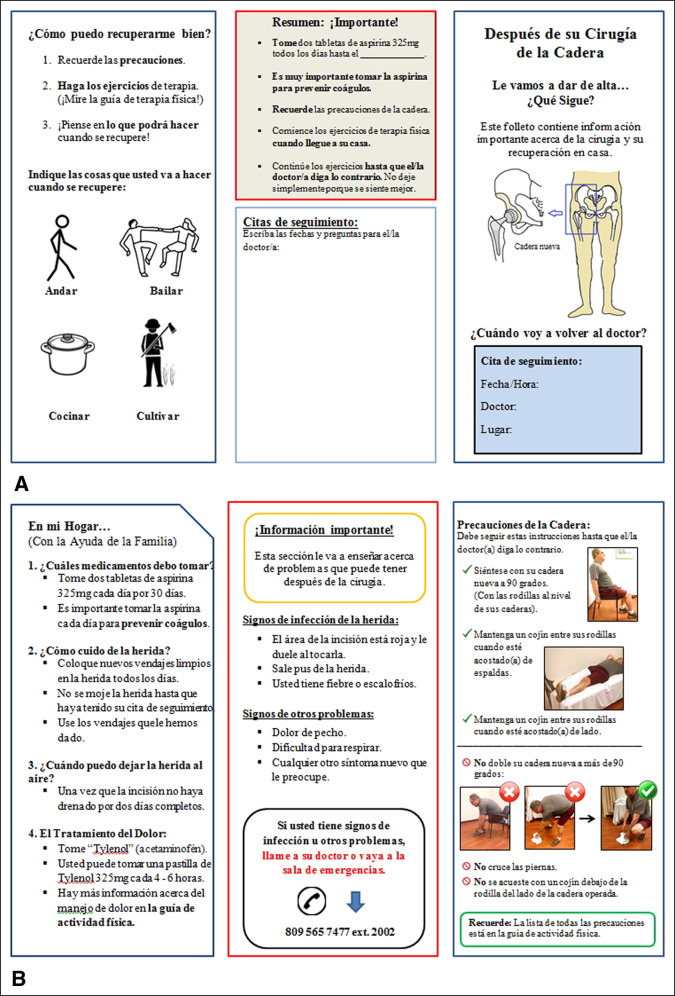
**A** and **B**, Illustration showing revised total hip replacement (THR) discharge instructions with new interactive pamphlet format.

The readability for all the original patient education materials was reduced by at least two grade levels. All the new materials had a readability level of fifth grade or less and superior suitability (Table 2). For comparison, we provide examples of the original patient discharge instructions materials (Figure 2) and the new patient discharge materials with an interactive pamphlet layout (Figure 3, A and B).

**Table 2:** Readability and Suitability Scores for Original and Revised Education Materials

Resource	Readability	Suitability
Original Physical Activity Guide THR (Spanish)	7th grade	45%
Revised Physical Activity Guide THR (Spanish)	5th grade	92%
Original Physical Activity Guide TKR (Spanish)	8th grade	—*
Revised Physical Activity Guide TKR (Spanish)	5th grade	—
Original THR Discharge Materials (Spanish)	8th grade	50%
Revised THR Discharge Materials (Spanish)	4th grade	93%
Original TKR Discharge Materials (Spanish)	8th grade	—
Revised TKR Discharge Materials (Spanish)	4th grade	—
THR FAQ (Spanish)	3rd grade	90%
TKR FAQ (Spanish)	3rd grade	—

Interpretation of Suitability ratings (SAM scale)	
70% to 100%	Superior material
40% to 69%	Adequate material
0% to 39%	Not suitable material

FAQ = frequently asked questions; SAM = suitability assessment of materials; THR = total hip replacement; TKR = total knee replacement

* SAM scores for the TKR resources were not calculated because they were developed based on the same template and principles as the corresponding THR resources.

## Discussion

To our knowledge, this is the first rigorous assessment of patient education materials for the surgical brigade setting. We undertook a systematic evaluation of the readability and suitability of existing patient education materials using the Fry readability graph and the SAM. The existing materials required an average readability level equivalent to the eighth grade and scored poorly on the SAM because of a combination of deficiencies in content, literacy demand, graphics, layout, learning stimulation, and cultural appropriateness. After consulting with brigade providers, we developed and evaluated new patient education materials using an evidence-based approach. These materials were revised to achieve appropriate readability levels and improved suitability. Pilot testing with patients from the target population suggested that the new materials were appealing and appropriate and guided additional revision based on patient feedback.

Common impediments to patient-provider communication such as language barriers, variable levels of patient education and health literacy, and time constraints on surgeons are often magnified during international surgical brigades. In many cases, no rehabilitation facilities exist to extend care and in-home physical therapy is not available. Surgical teams depart the country days after finishing the last procedure, and patients may live in rural areas far from emergency medical care.^36,37^ Adequate patient education has the potential to mitigate some of these limitations or prevent complications after the surgical team has departed. For example, implementation of a standardized patient education program combining pictographs with instructions in the local language lowered postoperative infection rates during a cleft palate surgical brigade in India.^56^ Additional research is needed to determine optimal modes of patient education and the effect of patient education on outcomes in these unique settings. Future efforts may even include electronic versions of materials for regions where patients have cell phone service.

We hypothesized that existing patient education materials were not optimal for the patient population served by the brigade, and our readability analysis confirmed this hypothesis (Table 2). The readability level of existing materials was too high for nearly half of the patients in our convenience sample who reported no secondary school education (Table 1). This sample is representative of the larger patient population served by the brigade.^57^ Discrepancies between the level of patient educational attainment and readability requirements of educational materials provided to them are not unique to our brigade.^40,58,59,60,61,62^ Nearly half of the US population reads at the eighth-grade level or below,^39^ and an abundance of studies have demonstrated that online health information resources available in English and Spanish for a variety of topics require reading skills above those of most US adults.^39,45,62,63,64,65^ Our study represents the first formal demonstration of a similar problem in the surgical brigade setting and is likely shared by other programs given the lack of standards for patient education during brigades.^3,6^

Patient education materials should always be designed with input from the intended audience because cultural appropriateness is a key determinant of the acceptability of health education material.^49,50,54^ We used published resources on designing health information materials for Latino/a audiences^51,54^ and lessons from prior TJA brigade research^55,57^ to guide initial development and then solicited feedback from patients. Our discharge materials emphasize the importance of family relationships in postoperative support and build trust by encouraging patients to record problems or questions to review them later with their provider. Interviews with patients indicated that the majority approved of the materials and would be comfortable interacting with and using them.

Despite measurable improvements, the new patient education materials did not meet the needs of every brigade patient interviewed. Some patients were unable to correctly answer comprehension questions after reading or listening to the information during interviews. This may be because of a combination of limited literacy and the challenging nature of certain topics. At least 20 percent of the patients interviewed were illiterate, and more than 50% had not graduated from secondary school. Patients struggled with questions regarding the time line for safely resuming sexual activity after surgery and expectations of pain after physical therapy exercises. These results from the comprehension questions allowed brigade staff to adjust their modes of patient training, incorporating more verbal education with recall and spending more time on difficult or nuanced topics. Tiered versions of educational resources that incorporate pictographs in lieu of complex language may be one solution to this problem.^39,59,60,66^ Patients with limited literacy should be identified so that extra time can be spent conveying important information. Failure to do so limits patients' health literacy and may reinforce existing health disparities.

Several important limitations of our study should be noted. We were unable to test the patient education materials with the target patient population during the initial development process, but this may be the case for many organizations attempting to develop surgical brigade patient education materials. We demonstrated methods to maximize suitability before testing on the ground in the host country. Another limitation of our study is the demonstrative rather than exhaustive nature of our suitability assessments for TJR resources. We were not able to compare our education materials with those used by other brigades, but we encourage other programs to examine their own practices. Finally, although its authors validated the SAM using the input of 172 healthcare providers from several cultures,^49^ it remains a relatively subjective measure of suitability. However, the SAM tool is freely available, is user-friendly, and provides a helpful metric to systematically improve health information materials before testing with patients. To minimize bias, we recruited an independent reviewer for SAM scoring who was not associated with this study.

## Conclusion

International surgical brigades provide care for thousands of patients every year,^3,4^ addressing a portion of the global burden of surgical disease.^42,43,67^ However, it is essential to acknowledge and guard against the potential pitfalls of temporary surgical platforms including the lack of adequate patient education. In addition to monitoring outcomes and ensuring follow-up care, brigades must provide personalized, compassionate, and appropriate surgical care. An essential component is patient education that empowers them to take charge of their recovery after the surgical team has departed.
